# High dose statin prophylaxis in cardiopulmonary bypass related surgery: clinical utility

**DOI:** 10.1186/s13019-017-0582-8

**Published:** 2017-03-31

**Authors:** Yie Roei Chee, R William G Watson, James McCarthy, Jehan Zeb Chughtai, Lars Nölke, David G Healy

**Affiliations:** 1 Cardiothoracic Surgery, Mater Misericordiae University Hospital, Eccles Street, Dublin 7, Ireland; 2 Conway Institute of Biomolecular and Biomedical Research, University College Dublin, Belfield, Dublin 4, Ireland

**Keywords:** Statin, Cardiopulmonary bypass, End-organ injury

## Abstract

**Background:**

Previous studies from our group demonstrated the anti-inflammatory properties of statins on cardiopulmonary bypass (CPB), through inhibition of neutrophil transendothelial migration. We sought to determine the utility of preoperative statin on patients undergoing cardiac surgery, to investigate any moderating effects on the systemic inflammatory response (SIRS) with CPB, and to evaluate any clinical impact on our patients.

**Methods:**

This is a prospective, randomised controlled trial with national regulatory body approval. Eligible patients were already on oral statin therapy. They were then randomly assigned to either investigation arm (*n* = 15, atorvastatin 80 mg for 2 weeks before surgery) or control arm (*n* = 15, no change to current statin therapy). Blood and urine samples were collected at 3 timepoints. Postoperative clinical measures were documented.

**Results:**

Patients in the investigation arm have significantly lower troponin level (*p* = 0.016), and lower level of urine neutrophil gelatinase-associated lipocalin (NGAL; *p* = 0.002); thus demonstrating a lesser degree of cardiac and renal injury in these patients. Higher level of Interleukin-8 (IL-8) at baseline (*p* = 0.036) and 4 h post cross-clamp removal (*p* = 0.035) in the investiation arm. A similar trend is also observed in Matrix Metalloproteinase-9 (MMP-9; *p* > 0.05). There were however no differences in clinical outcomes.

**Conclusions:**

Maximizing the dose of statin in patients waiting for cardiac surgery has measurable biological effects. There is evidence of less cardiac and renal damage. The use of preoperative statins and in particular, high dose preoperative statin therapy, may prove a useful new tool for optimal preparation of patients for cardiac surgery.

**Trial registration:**

EudraCT no. 2012-003396-20. Registered 05 November 2012

## Background

Cardiac surgery is known to provoke a vigorous inflammatory response. This is at least attributable to the use of cardiopulmonary bypass (CPB). The mechanisms by which CPB activates the inflammatory response included the direct ‘contact activation of the immune system following blood contact with the foreign surface of the apparatus, ischaemic-reperfusion injury [1] and the presence of endotoxaemia [2, 3]. These lead to the increase in humoral and cellular response, which ultimately leads to the development of systemic inflammatory response (SIRS). Once initiated, the SIRS is maintained by a number of proinflammatory mediators including interleukin-1 (IL-1), interleukin-2 (IL-2), interleukin-6 (IL-6), interleukin-8 (IL-8) and tumor necrosis factor-alpha (TNF-α) [4]. These in turn, facilitate the activation of neutrophil. The activated neutrophils release reactive oxygen species (ROS), causing damage to cellular membranes, stimulate leukocyte activation, chemotaxis and leukocyte-endohelial adherence [5]. These activated neutrophils, once migrated beyond the basement membrane through to the extracellular matrix (ECM) to the point of insult, will ultimately leads to tissue damage and multiple organ dysfunction. The development and use of new strategies aimed at prophylactically attenuate the systemic inflammatory response, and reducing postoperative morbidity following cardiopulmonary bypass related surgery, is of great importance.

Statin, a selective, competitive inhibitor of HMG-CoA reductase (3-hydroxy-3-methyl-glutaryl-CoA reductase), is recognised for its cholesterol lowering abilities. The use of statin is indicated in patients with hypercholesterolaemia as well as primary and secondary prevention of cardiovascular disease. There are, however, increasing evidences to suggest that statin also exerts a choleterol-independent, anti-inflammatory property [6]. The ‘pleiomorphic’ effects of statins have been reported in numerous acute and chronic inflammatory diseases [7, 8].

Previous in vivo and in vitro works from our group highlighted the protective properties of statin against inflammation. We showed that statins alter neutrophil migration via the Rho pathway [9], and further demonstrated the impact of high-dose statin therapy (≥40 mg) in transendothelial neutrophil migration [10]. In our recent in vivo study, we identified that high-dose statin (≥40 mg) attenuates MMP-9 production in the setting of CPB associated surgery. As yet, however, our studies have not shown a clinical benefit in patient outcomes. The defined clinical endpoints such as duration on mechanical ventilation, length of ITU and hospital stay are heavily influenced by external factors such as individual clinical practice, the availability of convalescence facility to facilitate patient discharge. It is therefore difficult to attribute any true meaning to any observed difference between patient groups.

This study aims to assess the optimal dose of pre-operative statin therapy in reducing end-organ injury and improved clinical outcome post CPB. CPB serves as an ideal model for analysis of ischemic-reperfusion injury as there is a definite start and end point. The cytokines chosen for this study have all been shown in previous studies to have an adverse effect on end-organ injury. By using more organ specific markers of injury, we aim to discover more subtle differences between our patient groups, which are less influenced by external factors.

## Methods

This prospective study included 30 patients who underwent CPB related surgery from March 2013 to May 2014 at the Mater Misericordiae University Hospital. The study protocol was approved by the Irish Medicine Board (IMB) and hospital ethics committee. Informed consent was obtained from each of the patients. We included patients who underwent elective surgery. Eligible patients were already on a statin therapy (dose ≤ 40 mg). Patients are either on Atorvastatin or Simvastatin as these are the two most commonly used statin therapy in our practice and both are similar in the molecular structure and efficacy. We excluded those on Rosuvastatin due to the differences in its potency as compared to Atorvastatin and Simvastatin. We excluded patients who had undergone emergency surgery, revision surgery, use of an intra-aortic balloon pump (IABP) preoperatively; patients with underlying infection disease or other significant disease such as chronic inflammatory disease, chronic liver disease, chronic kidney disease and malignancy; patients who were being treated with corticosteroids or anti-inflammatory or immunosuppresants. We performed a power analysis after the initial 10 patients were recruited. This demonstrated a total number of 30 patients (15 in each arm) were needed to adequately power the IL-8 and MMP-9 analysis. A clinical end-point was not chosen, as it would involve a large number of patients, which would require multicentre perhaps international cooperation. Our purpose is to lay the groundwork for such a study.

The 30 patients included in the study were randomized into 2 study arms: control arm (*n* = 15) which constituted patients who would continue their initial statin therapy (≤40 mg); and an investigation arm (*n* = 15) composed of patients who received atorvastatin 80 mg for at least 2 weeks before surgery. The block randomization is done using random allocation cards placed in a sealed envelope generated by an independent person unrelated to the study, picked by the investigator, to reveal the study arm in which the participant is allocated to. The duration of 2 weeks at which patients in the investigation arm received atorvastatin 80 mg is based upon our previous study, which demonstrated that neutrophil migration is attenuated following 2 weeks exposure of high-dose statin [10]. After surgery, these patients would return to their initial dosage of statin therapy.

### Surgical procedure and postoperative care

Patients undergoing cardiac surgery had a consistent anesthetic technique (propofol, fentanyl, pancuronium). Standard monitoring techniques (electrocardiogram, central venous and arterial catheter, urinary catheter, temperature probes) were used in all these patients. No patient received steroids before or during the operation.

All operations were performed through a median sternotomy. Sodium heparin (300 U/kg) was administered intravenously before CPB to achieve ACT ≥470 s. Standard cannulation technique (cannulation of the ascending aorta and the right atrium) was performed. Standard CPB circuit was used in all patients. This consists of a membrane oxygenator (Cobe, Cardiovascular, Sorin Biomedical, Gloucester, UK) and a roller pump (Computer Assisted Perfusion System, CAPS, STÖKERT, Sorin Biomedica, Gloucester, UK) primed with 1.0–1.2 l of crystalloid solution. Similar myocardial protection strategy was used in all. This consists of cold intermittent blood cardioplegia solution administered via the aortic root. Mild hypothermia was induced with the heat exchanger of the extracorporeal circuit and topical slush was used concomitantly.

At the end of the surgical procedures, the patients were transferred to the intensive care unit (ICU), where they remained sedated with morphine and midazolam (administered as needed). The patients remained intubated and connected to a mechanical ventilator set in synchronised intermittent mandatory ventilation (SIMV) with tidal volume 6–10 ml/kg, respiratory rate 12 breaths/min; the positive end-expiratory pressure set between 5 and 7 cm H_2_O; and fraction of inspired oxygen adjusted according to arterial blood gas analysis. The minimum mechanical ventilation time was 6 h; after 6 h, patients with stable haemodynamic, ventilation, oxygenation, and temperature conditions were extubated. Haemodynamic monitoring was maintained for at least the first 24 h of the postoperative period.

### Blood and urine sample collection, storage and analysis

White cell count, neutrophils and serum creatinine were measured at baseline (before the surgical intervention) and after surgery once the patients were stabilised.

Serum IL-8 and MMP-9 levels were determined in arterial blood samples at the following time points: at anaesthesia induction (reflecting patient’s baseline); 5 min post cross-clamp removal (reflecting CPB activation and reperfusion injury); 4 h post cross-clamp removal (reflecting CPB activation and ischaemic-reperfusion injury). Serum high-sensitive Cardiac Troponin (hs-cTnI) were determined in arterial bloods samples at anesthesia induction and 4 h post cross-clamp removal. Neutrophil gelatinase-associated lipocalin (NGAL) level were determined in urine samples at 4 h post cross-clamp removal.

The blood samples were collected via arterial line and immediately centrifuged at 1500 rpm for 15 min and stored at −80 °C until analysis. The urine samples were collected via indwelling urinary catheter and immediately centrifuged at 4 °C and 4000 rpm for 10 min and stored at −80 °C until analysis.

The IL-8 and MMP-9 were measured using the Mesoscale Discovery (MSD®), Multi Array Assay, ultra-sensitive kit (Maryland, USA), which is an enzyme-linked immunosorbent assay in conjuction with the MSD® SECTOR Imager SI 2400 automatic analyser. The hs-cTnI was measured using the Architect STAT Troponin-I assay (Abbott Diagnostics), which is a 2-step chemiluminescent microparticle immunoassay, in conjunction with the ARCHITECT iSystem optics ci8200. The NGAL was measured using the ARCHITECT urine NGAL assay (Abbott Diagnostics), which is a 2-step chemiluminescent microparticle immunoassay, in conjunction with the ARCHITECT iSystem optics ci8200.

### Statistical analysis

Statistical analysis was performed using the statistical software, SPSS 20.0 for windows. Each cytokine was measured at 3 timepoints. Significant differences in expression levels at each time point were examined using a Friedman test. This is the non-parametric alternative to the parametric one-way repeated measures analysis of variance (ANOVA) and was used due to the non-normality of cytokine expression. If significant differences were identified across time points using the Friedman test, a post hoc Wilcoxon test was used to identify which pairs of time point were significantly different in terms of cytokine expression. At each timepoint, the average cytokine expression for the control arm was compared to that of the investigation arm and significant differences were testing using a Mann Whitney *U* test. This is the non-parametric alternative to independent samples *t*-test. A *p* value of <0.05 was considered to be significant.

## Results

### Baseline patient characteristics

The clinical and operative characteristics were similar in the investigation arm and the control arm. Only the prevalence of diabetes were significantly higher in the investigation arm (*p* = 0.032) (Table 1). Liver function test at time of randomisation and after 2 weeks of treatment were similar in both groups, and no side effects were documented in patients (Table 2).

**Table 1:** Baseline, clinical and operative parameters of patients

Preoperative variables	Control arm (*n* = 15)	Investigation arm (*n* = 15)	*P*-Value
Age, year	64 (+/−2.1)	67 (+/−2.8)	0.442 N/S
Gender			0.152
Male	14 (93.3)	11 (73.3)	
Female	1 (6.7)	4 (26.7)	
Myocardial infarction			0.299
No	14 (93.3)	12 (80)	
Yes	1 (6.7)	3 (20)	
Diabetes mellitus			0.032
No	15 (100)	11 (73.3)	
Yes	0 (0)	4 (26.7)	
EF (%)			0.566
Good	9 (60)	11 (73.3)	
Fair	5 (35)	3 (20)	
Poor	1 (5)	1 (6.7)	
Euroscore II, %	0.87 (+/− 0.35)	1.11 (+/− 0.18)	0.101 N/S
Procedure			0.863
CABG	11 (73.3)	10 (66.7)	
Valve	0 (0)	1 (6.7)	
Combined	2 (13.3)	4 (26.7)	
Other	2 (13.3)	0 (0)	
CPB time, mins	108 (+/−9.8)	117 (+/−7.1)	0.271 N/S
Aortic cross-clamp, mins	92 (+/−7.9)	97 (+/−6.9)	0.722 N/S

*CABG* Coronary artery bypass grafting, *CPB* Cardiopulmonary bypass, *EF* Ejection fraction, *N/S* Not significant

Values are expressed as median (+/− SEM) or as number and percentage. *P* < 0.05 was considered statistically significant

**Table 2:** Comparison of liver function test at baseline and after treatment, and side effects related to statin

	Control arm (*n* = 15)	Investigation arm (*n* = 15)	*P*- Value
Liver function test (at baseline)			
Bilirubin, μmol/l	11 ± 1.1	12 ± 2.1	0.153
ALP, IU/l	80 ± 6.4	75 ± 5.3	0.858
ALT, IU/l	36 ± 6.6	22 ± 4.8	0.806
γGT, IU/l	24 ± 11.4	24 ± 8.1	0.403
Liver function test (after treatment)			
Bilirubin, μmol/l	10 ± 2.0	13 ± 2.7	0.916
ALP, IU/l	75 ± 5.0	87 ± 3.9	0.334
ALT, IU/l	26 ± 2.9	28 ± 3.8	0.676
γGT, IU/l	29 ± 6.1	23 ± 5.8	0.725
Side effects			
Rhabdomyolysis	0	0	--
GI Disturbances	0	0	--
Renal Impairment	0	0	--

### Postoperative patient course

The postoperative characteristics were similar between both arms. There were no renal injury or mortality in either group. There was a transient ischemic attack in the control arm, in which the patient made a complete recovery within 24 h after the onset of symptom (Table 3).

**Table 3:** Postopeative course of patients

Post operative variables	Control arm (*n* = 15)	Investigation arm (*n* = 15)	*P*-Value
Mechanical ventilation, hours	14 (+/− 0.9)	15 (+/− 4.3)	0.193
PaO2/FiO2 (at extubation)	44.3 (+/− 3.3)	43.4 (+/− 3.3)	0.289
PaO2/FiO2 (post extubation)	35.9 (+/− 6.2)	28.9 (+/− 3.7)	0.167
ICU stay, hours	21 (+/− 5.6)	18 (+/− 4.0)	0.510
Hospital stay, days	7 (+/− 0.6)	7 (+/− 0.9)	0.543
Acue kidney injury	0 (0)	0 (0)	N/A
Transient ischaemic attack	1 (6.7)	0 (0)	0.326
Mortality	0 (0)	0 (0)	N/A
Mortality at 1 Year	0 (0)	0 (0)	N/A

*FiO2* Fraction of inspired oxygen, *ICU* Intensive care unit, *PaO2* Partial pressure of oxygen, *N/S* Not significant

Values are expressed as median (+/− SEM) or as number and percentage. *P* < 0.05 was considered statistically significant

Both groups have similar respiratory index ratio pre extubation, however post extubation index was more favourable in the investigation arm.

### White cell count and neutrophil count

**Fig. 1:**
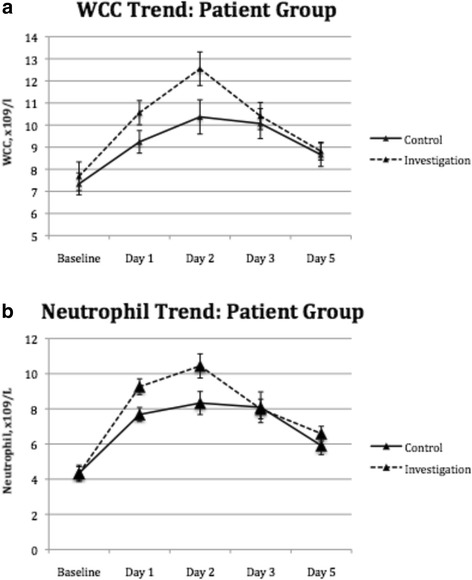
White cell count and neutrophils count over time, at baseline pre-operatively to day 5 post cardiopulmonary bypass related surgery in both the control arm (*n* = 15) and the investigation arm (*n* = 15) patients. **a** White cell count. **b** Neutrophil count. *Markers* indicate the median and *error bars* indicate the standard error of the mean. There was no statistically significant difference between the two groups in white cell count and neutrophil count

White cell count (WCC) and neutrophil count were collected on admission to day 5 postoperatively. Levels of WCC and neutrophil were elevated in both arms postoperatively, peaked at day 2 post-operation (Fig. 1). Level of WCC were higher in the investigation arm from baseline to day 5 postoperatively (*p* > 0.05) (Fig. 1a). Neutrophil counts were higher in the investigation arm from baseline to day 2 postoperatively but the patients in the control arm demonstrated higher level of neutrophil on day 5 post-operation (*p* > 0.05) (Fig. 1b).

### Interleukin-8 Levels

**Fig. 2:**
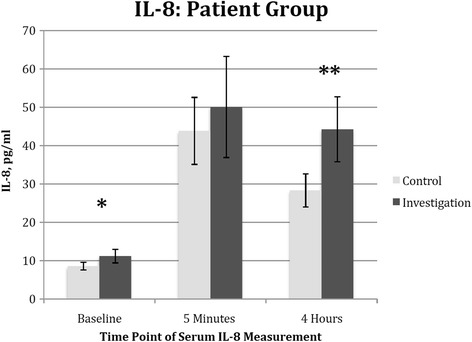
Effect of cardiac surgery with cardiopulmonary bypass on patient’s serum IL-8. Blood samples were collected from the patients preoperatively (as baseline), 5 min, 4 h post cross-clamp removal in both the control arm (*n* = 15) and the investigation arm (*n* = 15). *Columns* indicate the median and *error bars* indicate the standard error of the mean. There were statistically significant difference between the two groups at baseline, *p = 0.035 and at 4 h post cross-clamp removal, **p = 0.036

The serum IL-8 in the investigation arm was 11.2 +/− 1.8 pg/ml at baseline, vs. 8.6 +/− 1.0 pg/ml in the control arm, *p* = 0.036. An increased in IL-8 production was observed at 5 min after the release of the aortic cross-clamp but not statistically significant. At 4 h, the serum IL-8 in the investigation arm was 44.3 +/− 8.5 pg/ml vs. 28.3 +/− 4.3 pg/ml in the control arm, *p* = 0.035 (Fig. 2).

### Matrix metalloproteinase 9 levels

**Fig. 3:**
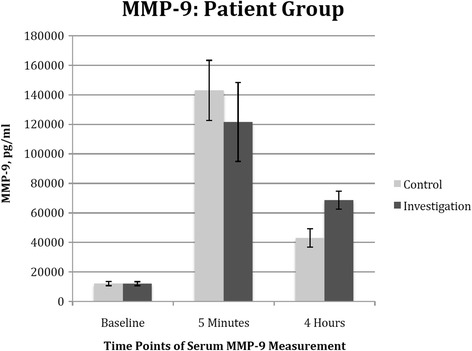
Effect of cardiac surgery with cardiopulmonary bypass on patient’s serum MMP-9. Blood samples were collected from the patients preoperatively (as baseline), 5 min, 4 h post cross-clamp removal in both the control arm (*n* = 15) and the investigation arm (*n* = 15). *Columns* indicate the median and *error bars* indicate the standard error of mean. There was no statistically significant difference between the two groups

The MMP-9 production was compared between both amrs. There was no statistical significant (*p* = 0.520) difference at any of the 3 time points between the two groups (Fig. 3).

### Level of serum creatinine

**Fig. 4:**
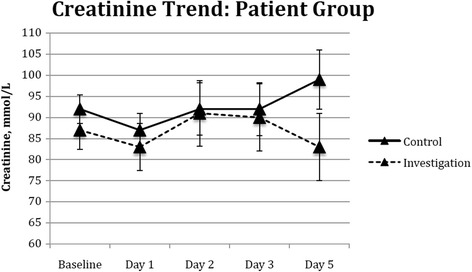
Effect of cardiac surgery with cardiopulmonary bypass on patient’s serum creatinine levels. Blood samples were collected from the patients on admission to day 5 postoperatively in both the control arm (*n* = 15) and the investigation arm (*n* = 15). *Markers* indicate the median and *error bars* indicate the standard error of the mean. There was no statistical significance noted between the two groups

Serum creatinine levels were collected on admission to day 5 postoperatively. Even though there was a trend of lower level of serum creatinine noted in the investigation arm, this is not statistically significant (Fig. 4).

### Level of urine neutrophil gelatine-associated lipocalin (NGAL) postoperatively

**Fig. 5:**
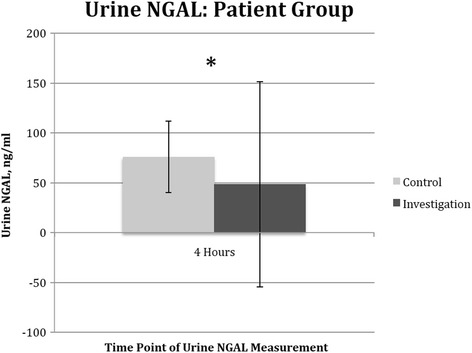
Effect of cardiac surgery with cardiopulmonary bypass on patient’s urine neutrophil gelatinase-associated lipocalin (NGAL). Urine samples were collected from the patients at 4 h post release of aortic cross clam in both the control arm (*n* = 15) and the investigation arm (*n* = 15). *Columns* indicate the median and *error bars* indicate the standard error of the mean. There were statistically significant difference between the two groups, *p = 0.002

Urine NGAL was measured in all patients postoperatively at 4 h post aortic cross-clamp removal. Urine NGAL was 75.9 +/− 35.9 ng/ml in the control arm; 48.4 +/− 102.8 ng/ml in the investigation arm, *p* = 0.002 (Fig. 5).

### Level of Troponin I

**Fig. 6:**
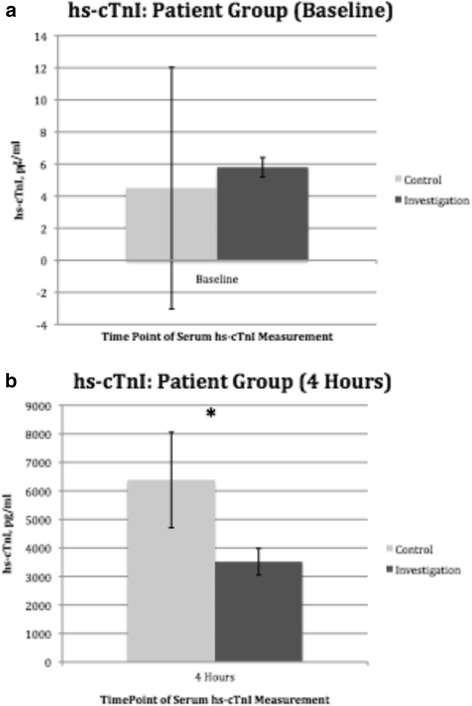
Effect of cardiac surgery with cardiopulmonary bypass on patient’s serum high sensitivity cardiac Troponin I (hs-cTnI) in both the control arm (*n* = 15) and the investigation arm (*n* = 15). **a** At baseline. **b** At 4 h post release of aortic cross-clamp. *Columns* indicate the median and *error bars* indicate the standard error of the mean. There were statistically significant difference between the two groups at 4 h post release of aortic cross-clamp, *p = 0.016

We analyzed the serum levels of hs-cTnI taken at two different times. The baseline hs-cTnI was similar between both groups (Fig. 6a). Hs-cTnI was 3516.1+/−465.2 pg/ml in the control arm; 6380.6+/−1672.5 pg/ml in the investigation arm, *p* = 0.016 (Fig. 6b). The hs-cTnIs were presented in two separate figures due to the vast difference in levels between the two timepoints.

## Discussion

The trauma of cardiac surgery, the use of CPB and the established ischemic-reperfusion injury, are responsible for the exaggerated activation of SIRS and its sequelae. The development of SIRS is due to an upregulation of both humoral and cellular responses. While much research has identified a wide variety of cytokines responsible and possible mechanism, little success was achieved in improving the clinical outcome associated with SIRS. Although the current standard clinical practice is very much focused on multi-organ supportive care in postoperative period, a preventative approach would have advantages.

We first demonstrated that neutrophils, which are thought to be primary mediator of SIRS, have potentially greater roles in cardiac transplantation than previously recognized, and suggested that blockade of the early allograft neutrophil infiltration might prevent subsequent lymphocyte recruitment and attenuate rejection [11]. Subsequently, the study showed that preoperative CD11b expression assessment might enable preoperative indication of patients who will mount an exaggerated and damaging neutrophil response to cardiac surgery [12]. We were then able to validate that the variation in individual neutrophil response influence the outcome of patients post cardiac surgery. These findings suggested that inhibition of neutrophil function and migration may be beneficial to improve clinical outcome [13].

There are increasing evidence that statins exert a cholesterol-independent, cardioprotective action. The ‘pleiomorphic’ effect of statins has been described in many acute or chronic inflammatory diseases such as atherosclerosis [14], sickle cell disease [15], sepsis [8]. A meta analysis of 91,491 patients following cardiac surgery [16], concluded that pre-operative statin therapy exerts substantial clinical benefit on early postoperative adverse outcomes. A separate systemic review [17] showed that prophylactic statin therapy can decrease the inflammatory response in patients undergoing CPB related surgery. We therefore initiated studies to evaluate the benefit of statin pre-treatment in attenuating the SIRS in CPB related surgery. We have focused however on the higher dose range. Indeed the control arm in this study is comparable to the treatment arm in other studies.

Maher et al. first demonstrated in an in vitro study, that statins reduce neutrophil migration towards the chemoattractant N-formyl-Methionine-Leucine-Phenylalanine (fMLP), and showed that statins blunt the neutrophil migration in a Rho dependent manner [9]. Kinsella et al. subsequently observed a difference in neutrophil migration, serum cholesterol and LDL level in healthy volunteers after two weeks of statin therapy [10].

In comparison to many studies published where patients were being classified as ‘with statin’ or ‘no statin’, our studies instead, divided the patients into doses of statin, i.e., high (≥40 mg) or low (<40 mg) dose when investigating the anti-inflammatory endpoints. In the observational study, we found that statins appeared protective against cardiac and renal injury in a dose dependent manner. We also showed that high-dose statin is associated with decreased MMP-9 production in patients undergoing CPB related surgery. While the results were very promising, clinical outcomes remained similar between both groups.

Recognizing the weaknesses of observational trials, we embarked on this prospective, randomised controlled trial. In this study, we showed that statin attenuates IL-8 production in a dose dependent manner; at 4 h post release of aortic cross-clamp. Interestingly, we observed a trend of higher neutrophil count in the investigation arm over the first 2 days post-operatively in spite of the lower IL-8 level. We proposed that this is probably due to the result of statin in attenuating neutrophil migration, resulting in higher level of extracellular neutrophil. In addition, we also demonstrated that statin is protective against end-organ injury as evident of a decreased cardiac and renal injury with lower levels of Troponin I and NGAL observed in the high-dose statin group.

Diagnosis of renal injury during CPB related surgery is not established in a timely fashion until significant damage has occurred. This is partly because of the lack of a clinically available early biomarker. Serum creatinine is used routinely in clinical practice, as it is the most widely available marker to diagnose renal injury. It is often not raised until >50% of renal function has already been lost. This is common at 48 h after the initial insult when the window period for early intervention is lost [18]. In our study, we found no significant change in patients’ serum creatinine from preoperative values to day 5 postoperatively, in both groups of patients.

We further evaluate the extent of renal injury, using urinary NGAL, a novel marker for renal injury. NGAL is currently being applied in clinical studies and is showing promising results [19]. NGAL can be detected as early as 2 h after CPB [20], and a single measurement of NGAL helps to distinguish acute kidney injury from normal function, pre-renal azotemia and chronic kidney disease [21]. There was a significant difference between patients in both groups of statin therapy at 4 h, which has not been described previously.

We found no significant difference in terms of the duration of mechanical ventilation, length of ITU or hospital stay between the groups. However, we did observe a trend of shorter duration of mechanical ventilation and length of ITU stay in the high-dose statin group. We would suspect that if this were to be repeated with higher numbers of patients we would reach significance.

There are a number of strengths and weakness to our study. Firstly, this is a prospective, randomised controlled trial, which provided a Level 1 evidence [22]. This is clearly superior compared to our previous studies, which are mostly observational, although we would also acknowledge that the fundamental design of this randomized controlled trial is based upon the findings from the previous studies. Secondly, other studies only compared the groups with statin or no statin. Our study however recruited patients who are already on a statin therapy, before randomized patients into the control or investigation arm. If we indeed compare patients with no statin versus high-dose statin therapy, a bigger effect might be expected. However ethical considerations of depriving patients of statins therapy completely prevented this. Our sample size was small. As those who were randomized to the investigation arm required a minimum of 2 weeks of high-dose statin, this restricts the recruitment of participants to only the elective patients. In our centre, 65% of the cardiac surgery performed in this time period was classed as urgent or emergency.

Looking forward, we have established that optimizing pre-operative statin therapy is associated with decreased end-organ injury in patients undergoing surgery. The ARMYDA-RECAPTURE trial, where 383 patients undergoing PCI were randomized to atorvastatin reload or placebo showed that reloading with high-dose atorvastatin improved clinical outcome of patients on chronic statin therapy undergoing PCI [23]. Additional studies are required to determine if this shorter incubation time will affect the immunological endpoints which we have looked at, and to establish whether reload with high-dose statin is associated with improved clinical outcome in patients.

This study has established the benefit of high-dose preoperative statin use in end-organ injury (cardiac and renal) in patients undergoing CPB related surgery. The inhibition of neutrophil migration is believed to be an important factor related to the SIRS induced by CPB. The use of statin, which is widely available at a relatively low price, is certainly an appealing option in improving patient outcome post CPB related surgery. This study has laid the groundwork for future studies looking into recruiting more patients with multicentre cooperation. As practicing clinicians, we advocate the use of high-dose preoperative statin therapy in patients undergoing CPB related surgery. Some of our consultants have indeed started to give high dose statin therapy to their patients scheduled to undergo CABG, while others have yet to commit to this practice. As mentioned, our continuous study is currently looking into determining if the result is replicable when patient is given shorter duration of high dose statin therapy, so that more patients can be recruited into the study.

## Conclusions

Maximizing the dose of statins in patients awaiting cardiac surgery has measurable biological effects. There is evidence of lesser cardiac and renal damage. The use of preoperative statins and in particular, high dose preoperative statin therapy, may prove a useful new tool for optimal preparation of patients for cardiac surgery. The research supports the routine use of statin therapy in patients undergoing cardiac surgery and highlights the specific importance of high dose statin therapy.

## Abbreviations

ACT: Activated clotting time
ANOVA: Analysis of variance
CD: Integrins
CPB: Cardiopulmonary bypass
ECM: Extracellular matrix
fMLP: N-formyl-Methionine-Leucine-Phenylalanine
HMG-CoA: 3-hydroxy-3-methyl-glutaryl-CoA
Hs-cTnI: High sensitivity cardiac Troponin I
IABP: Intra-aortic balloon pump
ICU: Intensive care unit
IL: Interleukin
IMB: Irish medicine board
ITU: Intensive therapy unit
LDL: Low-density lipoprotein
MMP: Matrix metalloproteinase
NGAL: Neutrophil gelatinase-associated lipocalin
PCI: Percutaneous coronary intervention
ROS: Reactive oxygen species
RPM: Revolutions per minute
SIMV: Synchronised intermittent mandatory ventilation
SIRS: Systemic inflammatory response syndrome
TNF- α: Tumour necrosis factor – alpha
WCC: White cell count
